# Defining clinical remission and clinically inactive disease in juvenile systemic lupus erythematosus (jSLE)

**DOI:** 10.1186/1546-0096-9-S1-O17

**Published:** 2011-09-14

**Authors:** Rina Mina, Laura Schanberg, Anne B Eberhard, Marisa Klein–Gitelman, Gloria Higgins, Karen Onel, Nora G Singer, Kathleen O’ Neil, Lori Tucker, Deborah Levy, Wajeeha Yousaf, Shannen Nelson, Michael Beresford, Ruben Cuttica, Graciela Espada, Angelo Ravelli, Alberto Martini, Edward Giannini, Hermine I Brunner

**Affiliations:** 1 Cincinnati Children's Med Ctr, USA; 2 Duke Children’s Hosp, USA; 3 Cohen Children’s Medical Ctr, USA; 4 Children’s Memorial Hosp, USA; 5 Nationwide Children’s Hosp, USA; 6 University of Chicago Comer Children‘s Hosp, USA; 7 MetroHealth Medical Center, OH, USA; 8 Children’s Hosp at Oklahoma University, USA; 9 BC Children’s Hosp, Canada; 10 The Hospital for Sick Children, Canada; 11 University of Cincinnati, OH, USA; 12 Royal Liverpool Children’s NHS Trust, UK; 13 Hospital General de Niños Pedro de Elizalde, Argentina; 14 Hospital de Niños Dr. Ricardo Gutiérrez, Buenos Aires, Argentina; 15 University of Genova, Istituto G. Gaslini, Italy

## Background

An initial Delphi survey delineated key commonalities for a standard definition of clinical remission and inactive disease in jSLE. However, several additional clarifications were still required.

## Objective

To develop a definition and criteria of clinical remission and inactive disease in jSLE.

## Methods

A second international Delphi survey was conducted among pediatric rheumatologists. Consensus was set at 75%.

## Results

There were 210 respondents. Consensus was achieved regarding the key definitions under consideration (Table 1). Respondents also agreed that a) there should be at most one mild, non-limiting symptom; and b) there can be regular use of several systemic medications in clinical remission. There was no consensus on whether select laboratory tests could be abnormal, and whether regular use of non-steroidal anti-inflammatory drugs with clinical remission was permissible.

**Table 1:** Definition of Clinical Remission and Clinically Inactive Disease in JSLE

Construct	Time frame	Acceptable Use of Medications for Lupus				Consensus
			
		Cortico steroids	Immuno suppressives	Preventive medications	Medications to treat SLE damage	
**Clinically Inactive Disease**	Time-point	Yes	Yes	Yes	Yes	94% (156/166)
**Clinical Remission on Medication**	Time-period: ≥ 6 months	Yes	Yes	Yes	Yes	96% (159/166)
**Clinical Remission on Preventive Medication**	Time-period: ≥ 6 montHs	No	No	Yes	Yes	95% (154/162)
**Clinical Remission Off Medication**	Time-period: ≥ 12 months	No	No	No	Yes	86% (140/162)

## Conclusions

Consensus has been reached on the definition of ‘Clinical Remission’ and ‘Clinically Inactive Disease’ in jSLE. The results of the Delphi process will be used to guide the data-driven development of provisional criteria of clinical remission and inactive disease in jSLE.

